# Application of Concanavalin A during immune responsiveness skin‐swelling tests facilitates measurement interpretation in mammalian ecology

**DOI:** 10.1002/ece3.2211

**Published:** 2016-06-10

**Authors:** Barbora Bílková, Tomáš Albrecht, Milada Chudíčková, Vladimír Holáň, Jaroslav Piálek, Michal Vinkler

**Affiliations:** ^1^ Department of ZoologyFaculty of ScienceCharles University in PragueViničná 7128 44PrahaCzech Republic, EU; ^2^ Research Facility StudenecInstitute of Vertebrate BiologyCzech Academy of SciencesKvětná 8603 65BrnoCzech Republic, EU; ^3^ Institute of Experimental MedicineCzech Academy of SciencesVídeňská 1083142 20Praha 4Czech Republic, EU

**Keywords:** Concanavalin A, cytokine, ecoimmunology, evolutionary immunology, field immunoecological research, histology, leucocytes, PHA‐induced hypersensitivity, phytohemagglutinin, T‐cell‐mediated immunocompetence

## Abstract

The skin‐swelling test is a simple and widespread method used in field ecological research to estimate cellular immune responsiveness in animals. This immunoecological test is based on measuring the magnitude of tissue swelling response at specific times following subcutaneous application of an experimental pro‐inflammatory stimulant. In the vast majority of studies across vertebrate taxa, phytohemagglutinin (PHA) is used as a universal stimulant. Given the complexity of immune response activation pathways of PHA, however, interpretation of test results can be ambiguous. Goal of this study was to improve methodology of the skin‐swelling test to decrease this ambiguity. Here, we present an alternative protocol aimed at facilitating interpretation of skin‐swelling data for mammals. Based on previous evidence suggesting that mammalian T cells are readily activated by Concanavalin A (ConA) in vitro, we compared cellular immune responses in vivo to PHA and ConA as an alternative pro‐inflammatory stimulant in mice. We measured magnitude of tissue swelling and compared it with intensity of blood cell infiltration into tissue over a 72‐hour interval. Our results corroborate that PHA and ConA show important differences in both dynamics and response amplitude in rodents. ConA induces stronger swelling with a distinct leukocyte activity pattern and higher pro‐inflammatory cytokine (interleukin 6 [IL‐6] and interferon gamma[IFN‐γ]) expression than PHA during peak response (24‐h post‐treatment). Furthermore, unlike PHA, magnitude of swelling was positively associated with cellular activity (number of neutrophils infiltrating tissue) following ConA injection. We conclude that ConA is the more suitable stimulant for skin‐swelling tests in mammals. This is because of the molecular binding specificity in the two lectins, that is, ConA specifically activates T cells while PHA also triggers erythroagglutination. We propose that ConA be used in all future ecological testing in mammals as it exhibits better performance and its application facilitates immunological interpretation of skin‐swelling test results.

## Introduction

The capacity to mount an appropriate immune response in a given ecological context is a fundamental adaptive trait measured in ecological studies (Sheldon and Verhulst 1996). Unlike laboratory‐based research, ecological field research is highly dependent on the practical feasibility of methods in the field, that is, with limited instrumentation and material equipment. The skin‐swelling test is an undemanding immunoecological method allowing comparison of the pro‐inflammatory capacity of individuals directly in the field (Smits et al. 1999; Kennedy and Nager 2006; Vinkler et al. 2010). The test is cheap, easy to perform and, to a great extent, taxonomically unspecific. As a result, the skin‐swelling test is the most commonly used method for measuring pro‐inflammatory immune responsiveness in animals. Following injection of a pro‐inflammatory stimulant into the hypodermic tissue of an investigated individual, infiltration of white blood cells is triggered, resulting in tissue swelling (Smits et al. 1999). The most frequently used pro‐inflammatory stimulant is phytohemagglutinin (PHA), a lectin isolated from the common bean *Phaseolus vulgaris* (Smits et al. 1999; Kennedy and Nager 2006; Ando et al. 2014). The PHA‐induced skin‐swelling test was originally developed as a clinical immunodeficiency test in human medicine (Bonforte et al. 1972) and veterinary science (Goto et al. 1978; Cheng and Lamont 1988). As then, it has been broadly applied in immunological and immunoecological studies in amphibians (Brown et al. 2011), reptiles (Finger et al. 2013), birds (Adelman et al. 2014; Tollington et al. 2015), and various mammal species, including rodents (Goüy de Bellocq et al. 2006a, 2007; Xu and Wang 2010; Merlo et al. 2014a,b; Zhang and Zhao 2015), bats (Turmelle et al. 2010), red deer (Fernandez‐De‐Mera et al. 2006, 2009), pig (Ekkel et al. 1995), wild boar (Jaroso et al. 2010), and cow (Hernandez et al. 2005).

The mechanisms behind PHA‐stimulated activation of inflammatory response has mostly been investigated in birds, where a relationship between swelling magnitude and intensity of cellular activity has frequently been observed (Martin et al. 2006; Vinkler et al. 2010, 2014). In contrast, PHA‐induced inflammation has rarely been studied in other animals, with no significant relationship found between swelling magnitude and tissue infiltration by leukocytes in mammals (Turmelle et al. 2010; Merlo et al. 2014b). As a result, interpretation of PHA skin‐swelling test results in mammals remains unclear, suggesting that PHA may not be the ideal stimulant for the method.

Based on its known biological activity, Concanavalin A (ConA), another plant lectin isolated from the jack bean *Canavalia ensiformis*, may represent a convenient alternative to PHA for ecological applications of the skin‐swelling tests (Weiss et al. 1987; Licastro et al. 1993). As with PHA, ConA is also commonly used as a stimulant of T‐cell activation and proliferation in vitro (Pialek et al. 2008; Akla et al. 2012; Ando et al. 2014; Chen et al. 2014). PHA and ConA are also both capable of triggering a pro‐inflammatory response in vivo in the absence of any natural antigen (Williams and Benacerraf 1972; Stadecke and Leskowit 1974; Ando et al. 2014). The mechanism of T‐cell activation differs between PHA and ConA, however, with PHA binding to both the *α*,* β*, and *γ* chains of the T‐cell receptor (PHA‐L subunit; Licastro et al. 1993) and to erythrocyte membrane compounds (PHA‐E subunit; Leavitt et al. 1977; Powell 1980) and ConA forming a specific bond with the T‐cell CD3 coreceptor only (Licastro et al. 1993). In PHA‐P, commercially available mixture of PHA‐L and PHA‐E subunits, both lymphocyte‐ and erythrocyte‐activating subunits are capable of potent nonspecific stimulation of inflammation when applied in vivo (Vinkler et al. 2010). Unlike PHA proteins, all ConA subunits are identical, that is, they display identical binding specificity (Lopez‐Jaramillo et al. 2004). Previous experimental studies have demonstrated that ConA induces stronger activation in mammalian splenic cells (Williams and Benacerraf 1972) and different lymphocyte types (Jones 1973; Jacobsson and Blomgren 1974) than PHA in vitro. Based on these distinct modes of response activation, we hypothesized that ConA and PHA treatment would evoke in vivo immune responses that differ in both dynamics and intensity. This hypothesis is supported by the findings of Stadecke and Leskowit (1974), who described distinct dermal responses to ConA and PHA in guinea pigs *Cavia porcellus*.

**Figure 1:**
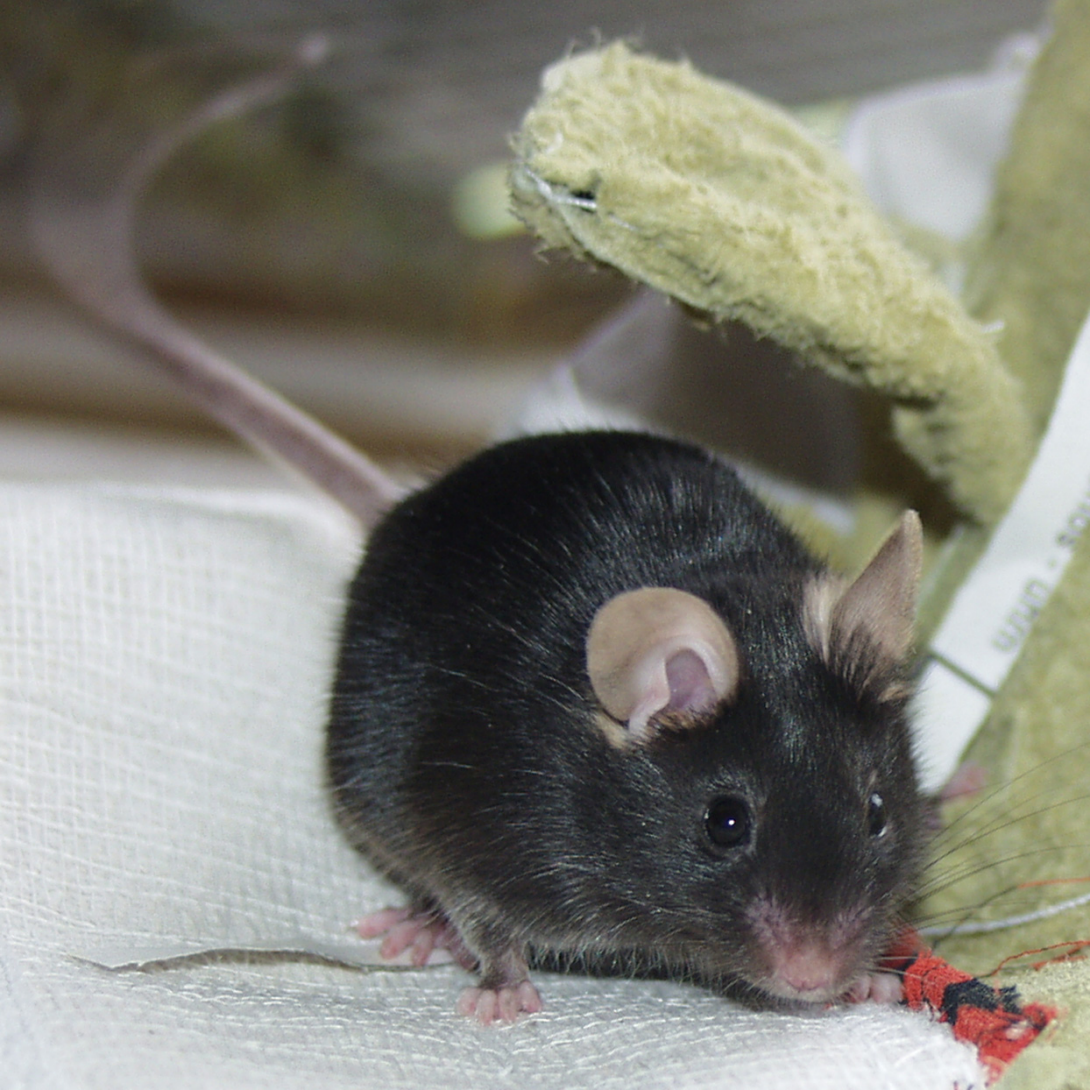
Mouse of the laboratory strain C57BL/6J. Although mouse inbred strains are typically investigated under laboratory conditions, they frequently serve as an ideal animal model for optimization of methods also in ecological studies (Weissbrod et al. 2013).

Given that the mechanism of leukocyte activation is more clearly explained in the ConA test, this study aimed to introduce ConA as a pro‐inflammatory stimulant for application in vivo and discusses the advantages of using ConA in mammal skin‐swelling tests. In order to provide data allowing improvement of the technique, we compare the temporal dynamics of inflammation induced by both ConA and PHA in mice based on tissue swelling metrics, histologically quantified tissue infiltration, and pro‐inflammatory cytokine expression. We use laboratory inbred mice (Fig. 1) as model organisms for this study as their uniform genetic background and laboratory environment allow us to reduce complexity of factors affecting our results and, thus, elucidate the immunological mechanisms in background of the test. This, nevertheless, does not preclude applicability of our results to field ecological research (see e.g., Weissbrod et al. 2013). The physiological mechanism studied here is a general one, and we believe that every immunological approach has to be validated first in a simple model system before it is adopted for field research in complex environments.

## Materials and Methods

### Experimental animals

Thirty‐five male C57BL/6J (BL6) laboratory strain mice aged 44–121 days were used for the swelling‐histology assay, and additional 22 males were employed for assaying cytokine expression during peak swelling response. Both treatment groups were age‐balanced. All males were obtained from the barrier‐free breeding facility at the Institute of Vertebrate Biology, Czech Academy of Sciences in Studenec, Czech Republic. The mice were housed individually under standard laboratory conditions as described in Pialek et al. (2008). In short, mice were kept in 30 × 15 × 15 cm plastic cages with permanent access to pelleted food (ST1; VELAZ, Prague, Czech Republic) and tap water. A 14:10 photoperiod was maintained, with lights on between 6:30 am and 8:30 pm.

### Experimental design

The 35 mice were divided into seven treatment groups (five individuals per group). The thickness of the left and right footpad was measured three times in each individual using a pressure‐sensitive digital thickness gauge with an accuracy of 0.01 mm (Mitutoyo 547‐301, Mitutoyo Corporation, Kanagawa, Japan) and repeatability of *r* = 0.85. A 0.025 mg dosage of ConA type IV (product No. C2010; Sigma‐Aldrich, St Louis, MO) dissolved in 20 μL of sterilized Dulbecco's phosphate‐buffered saline (DPBS; product No. D5652; Sigma‐Aldrich) was then intradermally injected into the hind left footpad of all experimental animals. At the same time, an identical dosage of PHA‐P (product No. L8754; Sigma‐Aldrich) in DPBS was injected into the hind right footpad. In the control mice (*n* = 5), the hind left footpad was injected with 20 μL of pure DPBS and the hind right footpad left untreated. In order to minimize experimental and temporal variation, all individuals were treated by the same experimenter over the same period, that is, between 4 pm and 8 pm. After treatment, all mice were returned to their cages and left undisturbed until response measurement. Swelling response magnitude was measured after six distinct time periods in different groups, that is, after 3 h (*n* = 5), 6 h (*n* = 5), 12 h (*n* = 5), 24 h (*n* = 5), 48 h (*n* = 5), and 72 h (*n* = 5). In the control mice, thickness change was measured in both paws 24 h after treatment. As above, we measured tissue thickness three times with an accuracy 0.01 mm (repeatability of *r* = 0.99). The skin‐swelling response was later calculated as the average footpad thickness following treatment minus the average thickness before injection. Each individual was then euthanized through cervical dislocation. A necropsy sample of treated tissue was taken from each footpad and fixed in 10% buffered formalin.

The research was approved by the Ethical Committee of the Institute of Vertebrate Biology, Czech Academy of Sciences (Permit No. 27/2007) and was carried out in accordance with the current laws of the Czech Republic and EU.

### Histology

As the histology protocol has previously been described in detail by Vinkler et al. (2010), we describe it here only briefly. The necropsy tissue samples collected from each footpad were stored in 10% buffered formalin at +4°C until processing at a commercial histological laboratory (BIOLAB Praha Laboratory, Prague, Czech Republic). Here, they were embedded in paraffin, sectioned, and stained with hematoxylin and eosin. Three sections were made from the metatarsus of each hind foot sample. In each section, three independent sites were photographed (i.e., nine photographs per sample) under a 40× objective magnification (Olympus BX51 microscope fitted with an Olympus DP71 digital camera, Tokyo, Japan; QuickPHOTO Industrial imaging software version 2.3; Promicra, Prague, Czech Republic). Cells were counted within a 0.0025‐mm^2^ frame using IMAGEJ 1.44p software (National Institutes of Health, Bethesda, MD) (http://rsbweb.nih.gov/ij). Five cell categories were recognised: lymphocytes, monocytes, neutrophils, basophils, and erythrocytes. Thus, we obtained a total cell count as the sum of all cells and individual cell type counts as the number of individual cells recalculated per 0.01 mm^2^ of tissue section.

### Cytokine gene expression assay

Quantitative polymerase chain reaction was used to estimate the expression of genes encoding pro‐inflammatory cytokines interleukin 6 (IL‐6) and interferon gamma (IFN‐*γ*) in 22 experimental males using footpad samples from control/PBS‐ (*n* = 11) or ConA/PHA‐treated mice (*n* = 11). These individuals were different from those used for the histological examination but were injected the same treatment doses as described above and measured 24 hours after the treatment. Palmar tissue was excised and frozen at −80°C in microtubes containing 500 μL of TRI Reagent (Molecular Research Center, Cincinnati, OH). Total RNA was isolated using TRI Reagent with Polyacryl Carrier (Molecular Research Center) according to the manufacturer's instructions. One μg of the total RNA was pretreated using deoxyribonuclease I (Promega, Madison, WI) and subsequently used for reverse transcription. The first‐strand cDNA was synthesized using M‐MLV reverse transcriptase and random primers (Promega) in a total reaction volume of 25 μL.

Quantitative real‐time PCR was performed on an iCycler (Bio‐Rad, Hercules, CA), using iQ SYBR Green Supermix (Bio‐Rad) as we have described elsewhere (Trosan et al. 2012). The following primers were used for the amplification: GAPDH (the housekeeping gene): 5′‐CCCAACGTGTCTGTCGTG‐3′ (sense), 5′‐CCGACCCAGACGTACAGC‐3′ (antisense), IL‐6: 5′‐GGGCAAGATGATGCCAAA‐3′(sense), 5′‐TTGTGATGACAGTTTGGTGAGTC‐3′ (antisense), IFN‐*γ*: 5′‐GGGTAACTGTGAATGTTCAATGG‐3′(sense), 5′‐GCTCAGAAACCCAGTTGCAT‐3′ (antisense). Each measurement was undertaken in duplicate. The PCR parameters were as follows: denaturation at 95°C for 3 min, then 40 cycles with denaturation at 95°C for 20 sec, annealing at 60°C for 30 sec, and elongation at 72°C for 30 sec. Fluorescence data were collected at each cycle after an elongation step at 75°C for 5 sec and were analyzed on the iCycler detection system (Bio‐Rad, Hercules, CA), version 3.1. A relative quantification model (∆*C*_t_) was applied to calculate the expression of the target gene in comparison with GAPDH which was used as an endogenous control.

### Statistical analysis

Given the small sample size available for each time point, nonparametric Wilcoxon rank‐sum test (with paired approach where observations were statistically dependent, that is, derived from the same individual) was used to test the equality of means. However, where sample size and data normality allowed (average cellular infiltration between PHA‐ and ConA‐treated footpads), we used the Student's paired *t*‐test to compare datasets. Normality of data distribution was tested using the Shapiro–Wilk test. Dependence of tissue swelling and cellular infiltration on time of treatment, age, and animal weight following PHA and ConA treatments were assessed with generalized linear models (GLM) using the identity link function (all full models and their overall significance in Table A2; Time 0 = control group of untreated footpads). A minimum adequate model (MAM; model with all terms significant; Crawley 2012) was obtained through backward elimination of terms from the full model. Structure and levels of significance of MAMs are shown in Table 1. Differences in cellular infiltration and tissue swelling between time points were later tested using Tukey's honest significant difference test. We used linear regression to test for a relationship between tissue swelling and cellular infiltration. For GLM and regression models, normality of model residuals was checked in all cases using the Shapiro–Wilk test. The standard error was calculated to assess the level of sample variation, and the significance level was set at *P* = 0.05. All statistical analyses were performed using the R statistical program (R core Team, 2014).

**Table 1:** Minimal adequate models (MAMs) and their levels of significance

	Minimal adequate model	Significant terms	*F*	df	*P*
MAM1	Swelling PHA ~ time		31.32	6/28	<0.001
MAM2	Swelling ConA ~ time + age		20.53	7/27	<0.001
		Time	23.28	6/27	<0.001
		Age	7.58	6/27	0.010
MAM3	Total cellular infiltration PHA ~ time		4.80	6/28	0.002
MAM4	Number of neutrophils PHA ~ time		3.64	6/28	0.008
MAM5	Total cellular infiltration ConA ~ time		16.69	6/28	<0.001
MAM6	Number of neutrophils ConA ~ time		18.79	6/28	<0.001
MAM7	Number of macrophages ConA ~ time + age		5.29	7/27	<0.001
		Time	3.74	6/27	0.008
		Age	20.11	6/27	<0.001
MAM8	Number of lymphocytes ConA ~ time + weight		4.85	7/27	0.001
		Time	2.62	6/27	0.038
		Weight	16.94	6/27	0.001

Partial effects of significant terms are shown in separate lines in those cases where MAM includes more than one significant variable. Swelling PHA/ConA = magnitude of the tissue thickness change after PHA/ConA treatment; number of lymphocytes/neutrophils/macrophages PHA/ConA = number of cells infiltrated into the tissue (per 0.01 mm^2^) after PHA/ConA treatment; time = time of the measurement after the treatment; age = age of an animal in days; weight = weight of an animal in grams.

## Results

### Swelling response metrics

**Figure 2:**
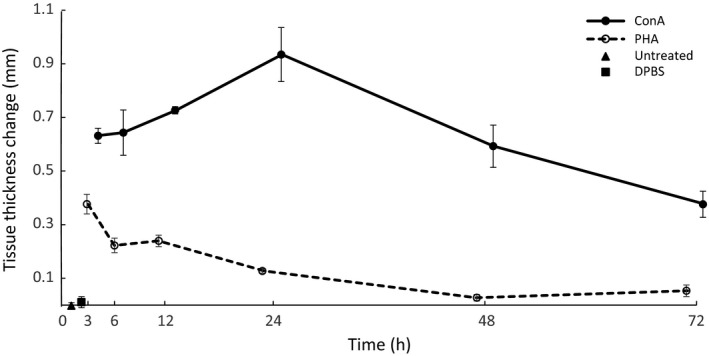
Temporal dynamics for tissue swelling (tissue thickness change) following ConA (solid line, filled dots) and PHA treatments (dashed line, open circles). At time point 0, the triangle indicates the untreated control and the square indicates the DPBS‐treated control. Error bars show standard errors of the mean.

There was no significant difference between thickness of left and right footpad before treatment (Wilcoxon paired test; *V* = 541.50, *P* = 0.407). Twenty‐four hours after stimulation, we observed no difference in footpad thickness between the unstimulated footpad and that injected with DPBS (Wilcoxon paired test; *V* = 4, *P* = 0.855). Likewise, there was no difference in thickness in the stimulated footpad before and 24 h after injection, indicating that DPBS does not stimulate tissue swelling (Wilcoxon test; *V* = 6, *P* = 0.813). In contrast, application of both PHA and ConA caused a measurable increase in footpad thickness caused by a swelling response (Wilcoxon paired test across all time points; ConA: *V* = 465, *P* < 0.001; PHA: *V* = 435, *P* < 0.001), with the mean magnitude of swelling greater when stimulated with ConA (Wilcoxon paired test; *V* = 0, *P* < 0.001). Both PHA and ConA caused a time‐dependent swelling response (GLM; PHA, significant term time: MAM1 in Table 1; ConA, significant terms age and time: MAM2 in Table 1), with a swelling response (tissue thickness significantly different from time point 0) measurable at 3, 6, 12, and 24 h after PHA application and at 3, 6, 12, 24, 48, and 72 h after ConA application (Tukey test; 3, 6, 12, and 24 h after PHA, *P* < 0.05; all time points after ConA, *P* < 0.05; Fig. 2). The temporal dynamics of response differed between stimulants, with maximum PHA swelling (0.377 ± 0.037 mm) observed at 3 h and maximum ConA swelling (0.934 ± 0.100 mm) observed 24 h after injection (Fig. 2; Table A1).

### Tissue cellular infiltration

Despite the lack of swelling response, DPBS treatment resulted in significant cell infiltration into tissue (Wilcoxon paired test of DPBS‐treated and untreated footpad; *V* = 25, *P* = 0.011). The increased tissue cellularity was mainly caused by infiltration of neutrophils into the DPBS‐treated tissue (Wilcoxon paired test, *V* = 23.5, *P* = 0.026), the number of other cell types remaining unchanged between stimulated and unstimulated tissue (*P* > 0.05 in all cases).

**Figure 3:**
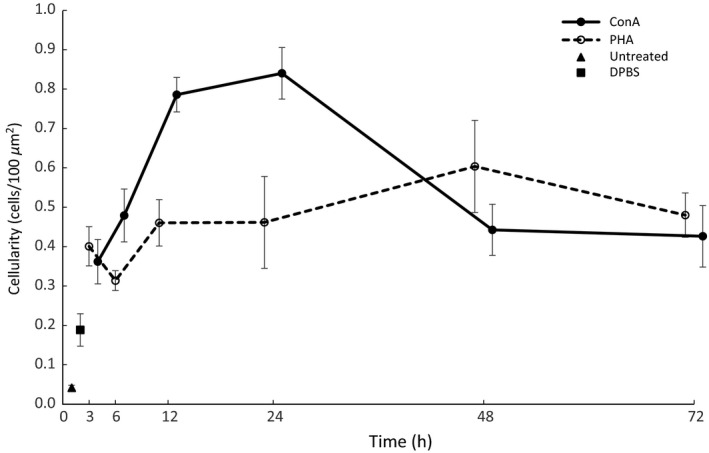
Temporal dynamics for total cellular infiltration into ConA‐ (solid line, filled circles) and PHA‐treated (dashed line, open circles) tissue. At time point 0, the triangle indicates the untreated control and the square indicates the DPBS‐treated control. Error bars indicate standard errors of the mean.

Injection of both PHA and ConA induced leukocyte infiltration into the tissue (Table A1). In PHA‐treated tissue, the total number of cells detected depended significantly on time after stimulation (GLM; significant term time: MAM3 in Table 1), with a significant difference in total cellular infiltration (compared with the control at time point 0) observable 12, 24, 48, and 72 h poststimulation (Tukey test; *P* < 0.020 in all cases), the difference at 3 h poststimulation being marginally nonsignificant (*P* = 0.056). Hence, there was no significant difference in tissue infiltration at peak PHA response (apparently 48 h following injection) and any other time point (Tukey test, in all cases *P* > 0.05), indicating a constant cell number in tissue during the immune response (Fig. 3). We also detected a significant effect of time after injection on infiltration of individual leukocyte types, neutrophils in particular (GLM; significant term time: MAM4 in Table 1), with a significant difference in neutrophil infiltration observable at 24, 48, and 72 h (Tukey test; *P* < 0.05 for all leukocyte types compared individually).

**Figure A1:**
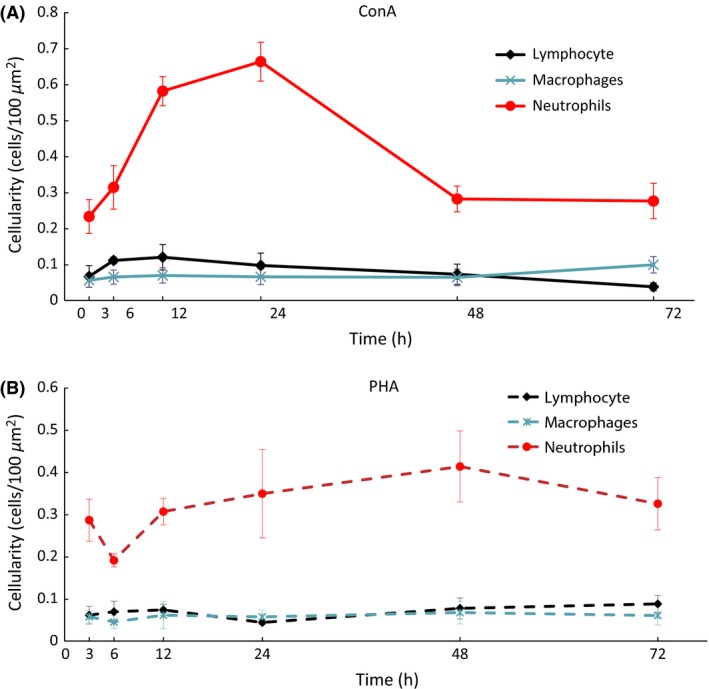
Temporal dynamics for the infiltration of individual cell types into tissue following (A) ConA and (B) PHA treatment. Individual cell types are differentiated by colour and symbol: neutrophils = red circles, lymphocytes = black diamonds, macrophages = blue crosses.

**Figure A2:**
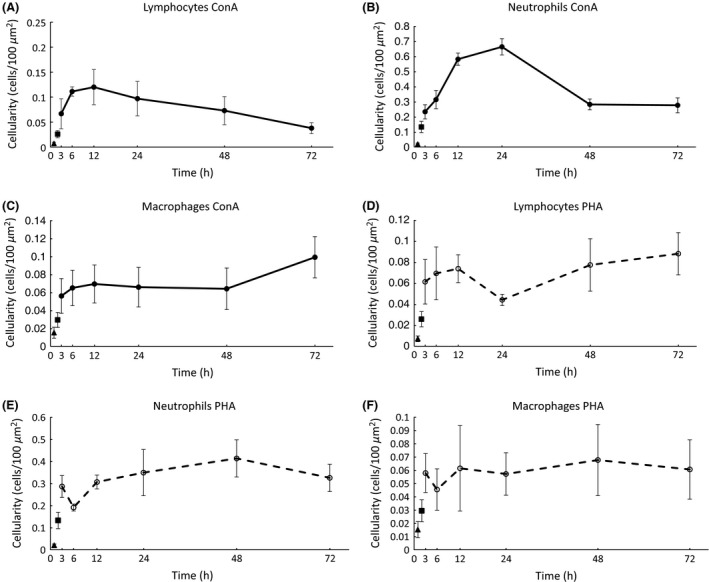
Temporal dynamics for the infiltration of individual cell types into tissue following ConA and PHA treatment: (A) infiltration of lymphocytes into ConA‐treated tissue; (B) infiltration of neutrophils into ConA‐treated tissue; (C) infiltration of macrophages into ConA‐treated tissue; (D) infiltration of lymphocytes into PHA‐treated tissue; (E) infiltration of neutrophils into PHA‐treated tissue; (F) infiltration of macrophages into PHA‐treated tissue. At time point 0, the triangle indicates the untreated control and the square indicates the DPBS‐treated control.

Total number of cells infiltrating tissue following ConA stimulation was also related to time (GLM; significant term time: MAM5 in Table 1), with a significant difference observable between the control (time point 0) and all time points, and between time points 12 and 24 h and all other time points (Tukey test; *P* < 0.038 in all cases). Unlike PHA stimulation, therefore, peak response (24 h after ConA injection) was significantly distinct from most other time points, that is, 3, 6, 48, and 72 h poststimulation (Tukey test; *P* < 0.003 in all cases; Fig. 3). We also observed significant time‐dependent changes in infiltration of individual leukocyte types, namely neutrophils (GLM; significant term time: MAM6 in Table 1, Figs. A1a, A2b), macrophages (GLM; significant terms time and age: MAM7 in Table 1, Fig. A1a, A2c), and lymphocytes (GLM; significant terms time and weight MAM8 in Table 1, Figs. A1a, A2a).

Phytohemagglutinin‐ and ConA‐injected tissue showed distinct cellular infiltration dynamics (Fig. 3), with differences most apparent with neutrophil infiltration (Figs. A1, A2). Despite such clear temporal patterns, however, we found no statistically significant difference in mean cellular infiltration between PHA‐ and ConA‐treated footpads (Paired *t*‐test; all cell types across all time points: *P* > 0.05).

**Figure 4:**
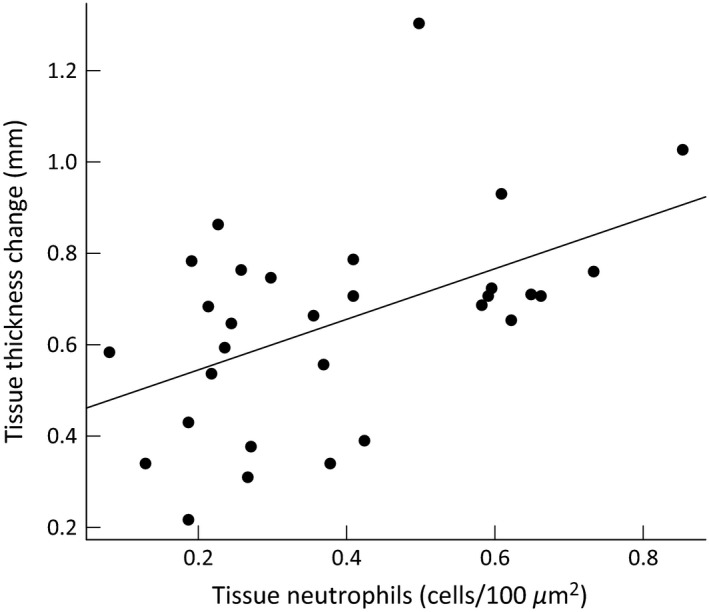
Relationship between neutrophil infiltration into tissue and magnitude of the swelling response (tissue thickness change) following ConA injection.

**Figure A3:**
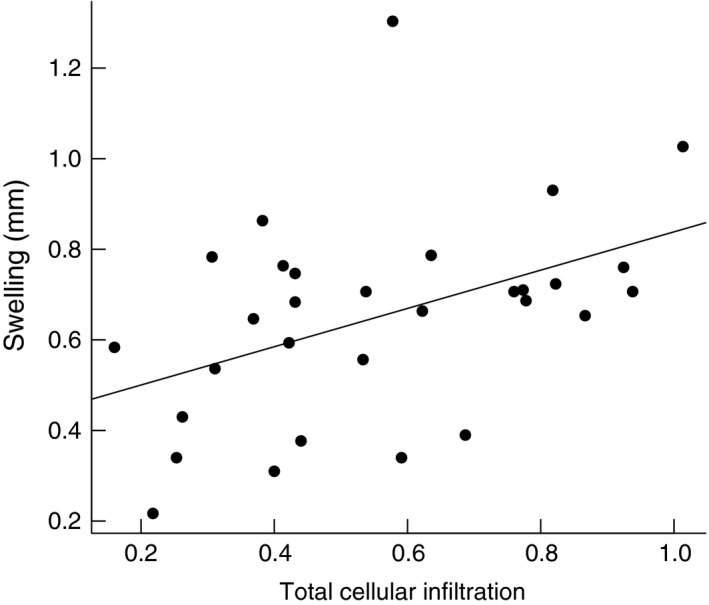
Relationship between total cellular infiltration and magnitude of tissue swelling following ConA treatment. The slope of the fitted line is significantly different from zero (*R* ^2^ = 0.20, slope = 0.42 ± 0.16, *F*_1,28_ = 6.90, *P* = 0.014).

In PHA‐stimulated tissue, we observed no relationship between magnitude of swelling and cellular infiltration (*P* > 0.05). In contrast, we observed a strong positive relationship between tissue swelling and number of cells infiltrating tissue in ConA‐stimulated tissue (*R* ^2^ = 0.20, slope = 0.42 ± 0.16, *F*_1,28_ = 6.90, *P* = 0.014; Fig. A3). This association was mainly due to a strong relationship between magnitude of swelling and number of infiltrating neutrophils (*R* ^2^ = 0.25, slope = 0.55 ± 0.18, *F*_1,28_ = 9.12, *P* = 0.005, Fig. 4). Neutrophils are the most abundant cell type in the swollen tissue (Table A1, correlation between total cell count and neutrophil count in ConA‐treated tissue is *r* = 0.977, *P* < 0.01).

### Cytokine expression during peak ConA response

**Figure 5:**
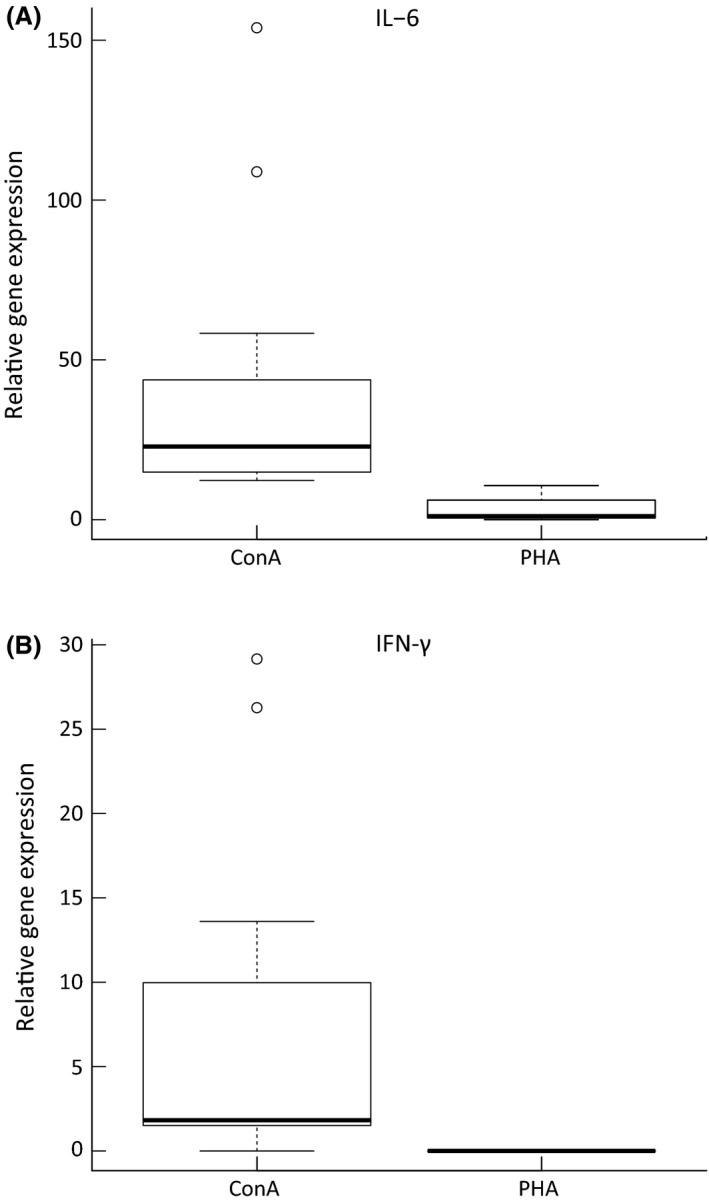
Expression of pro‐inflammatory cytokines IL‐6 (A) and IFN‐*γ* (B) in ConA‐ and PHA‐treated tissue. Expression was measured 24 h after treatment. Boxplots indicate the median (bold solid lines) and upper and lower quartiles (upper and lower box boundaries), with whiskers indicating maximum and minimum within 1.5 multiples of the quartile range and dots indicating outliers.

We observed no measurable expression of IL‐6 and IFN‐*γ* in the control (nonstimulated) or DPBS‐stimulated footpad samples. In contrast, we observed IL‐6 transcription in both PHA‐ and ConA‐stimulated tissues 24 h after treatment (Wilcoxon test; PHA: *V* = 110, *P* < 0.001; ConA: *V* = 121, *P* < 0.001), with IL‐6 expression significantly higher in the ConA‐stimulated tissue (Wilcoxon test; *V* = 66, *P* < 0.001; Fig. 5). Although we found upregulated expression of IFN‐*γ* in ConA‐stimulated tissue (Wilcoxon test; *V* = 115.5, *P* < 0.001), we detected no such expression in PHA‐stimulated footpads.

## Discussion

Both ecological field research and inference are highly dependent on the practical feasibility of the methods adopted. Compared to laboratory‐based research, there is a much greater requirement for technical simplicity with regard to instrumental equipment and materials. As the skin‐swelling test represents an undemanding immunoecological method allowing comparison of the pro‐inflammatory capacity of individuals directly in field, it is no wonder that it is commonly used for testing immune responsiveness in a diverse spectrum of free‐living animal taxa (Goüy de Bellocq et al. 2006b; Fernandez‐De‐Mera et al. 2008; Wiley et al. 2009; Brown et al. 2011; Finger et al. 2013). As a means of improving present methodology and facilitating interpretation of the results, we proposed ConA as an alternative skin‐swelling test stimulant and compared its activity with that of PHA. Our results demonstrate that ConA applied in vivo stimulates a stronger inflammatory response in mammals than PHA. Importantly, we also show that, unlike PHA, the swelling response triggered by ConA mirrors the pattern of cellular activity within the tissue. Knowledge of the relationship between swelling data and cellular activity is crucial for correct interpretation of the test's results.

Intradermal injection of both PHA and ConA lectins triggered significant in vivo tissue swelling and cellular infiltration compared to a control. As expected, we were also able to detect a measurable infiltration of blood cells (mainly neutrophils) into tissue in the DPBS‐injected controls, despite the lack of any swelling response. Cellular infiltration into DPBS‐injected tissue has also been observed in previous studies on birds (Martin et al. 2006) and mammals (Turmelle et al. 2010). This could be explained as a weak local immune response to damage to the footpad tissue caused by the needle. In agreement with this assumption, we observed only mild cellular infiltration into DPBS‐injected control tissue and no detectable upregulation in pro‐inflammatory cytokine (IL‐6 and IFN‐*γ*) expression.

Comparison of the two stimulants demonstrated clearly that ConA produces a stronger measurable swelling response than PHA in mice. In agreement with our results, Stadecker and Leskowitz (1974) reported that ConA induced stronger erythema in guinea pigs than PHA. These in vivo results agree well with the corresponding results of in vitro studies showing differences between ConA‐ and PHA‐triggered mitogenesis. It has been repeatedly demonstrated in mammals that ConA induces stronger proliferation of spleen (Williams and Benacerraf 1972) and thymic (Jacobsson and Blomgren 1974) T cells (Jones 1973) than PHA. It has also been suggested by Stobo and Paul (1973) that the two lectins may stimulate different subpopulations of T cells.

ConA‐ and PHA‐treated tissue showed distinct swelling response and cellular infiltration dynamics, both in our study of mice and the Stadecker and Leskowitz (1974) study of guinea pigs. Injection of ConA induces significant tissue swelling lasting between 3 and 72 h, with a maximum at around 24 h, while PHA‐induced swelling peaks earlier (3 h after treatment) and disappears more rapidly. The same swelling dynamics pattern following PHA stimulation has also been observed in other mammal studies. In Brazilian free‐tailed bats *Tadarida brasiliensis* (Turmelle et al. 2010), striped hamsters *Cricetulus barabensis* (Zhang and Zhao 2015), and Talas tuco‐tucos *Ctenomys talarum* (Merlo et al. 2014b), swelling response was greatest during the first measurement following injection (i.e., 6 h; no data available for 3 h after injection), following which it decreased. Interestingly, it appears that this pattern is taxon‐dependent as an opposing tissue swelling pattern has been described in birds (Martin et al. 2006), reptiles (Finger et al. 2013), and amphibians (Brown et al. 2011), that is, tissue thickness continued to increase in the 24 h after PHA injection.

Our results reveal that, while there is no relationship between tissue swelling and cellular infiltration in PHA‐treated tissue, the association is both positive and significant in ConA‐injected footpads, with maximum cellular infiltration following ConA injection (significant increase at 24 h; Fig. 3, Table A1) perfectly matching the peak swelling response and a clear mismatch between peak skin swelling (3 h) and cellular infiltration (apparently at 48 h but nonsignificant) after PHA treatment. A similar mismatch between swelling response and cellular infiltration in PHA‐injected tissue has also recently been described in other mammalian species (Turmelle et al. 2010; Merlo et al. 2014b). This suggests that, in mammals, a PHA‐triggered immune response measured as change in tissue thickness cannot be interpreted in terms of cellular activity within the tissue.

Neutrophils were the most abundant cells in both PHA‐ and ConA‐stimulated tissues, with lymphocytes and macrophages second most abundant. This is in full agreement with previous mammal studies (Turmelle et al. 2010; Merlo et al. 2014a). Although neutrophils, lymphocytes, and macrophages all increase in number in both PHA‐ and ConA‐injected tissues, only neutrophils infiltrating ConA‐stimulated tissue show a clear temporal dynamics pattern (Fig. A2). This pattern resembles that observed in other species (including nonmammalian species) following PHA application, with a rapid increase in neutrophil number 12–24 h after stimulation (Martin et al. 2006; Turmelle et al. 2010; Brown et al. 2011; Finger et al. 2013). In our study, peak ConA‐triggered neutrophil infiltration matched peak swelling response (Figs. 3, A2). This, together with the strong association between tissue cellular/neutrophil count and swelling magnitude in ConA‐treated footpads (Fig. 4), allows for biologically meaningful interpretation of skin‐swelling results measured 24 h after ConA injection.

In ConA‐treated tissue, lymphocyte number increased early and remained relatively stable over time, while neutrophil infiltration varied greatly over time but with a clear peak 24 h after injection (Figs. A1, A2). Hence, we propose that lymphocytes are activated by ConA at the beginning of the inflammatory response and that these lymphocytes later stimulate neutrophil infiltration, resulting in a measurable tissue swelling response.

High neutrophil and macrophage numbers in PHA‐ and ConA‐injected tissue suggests that the pro‐inflammatory response is mediated by Th17 and Th1 cells (Desmedt et al. 1998; Iwakura et al. 2008). This is partially confirmed by our results for cytokine expression, which show IFN‐*γ* upregulation in ConA‐stimulated tissue and IL‐6 expression in both ConA‐ and PHA‐stimulated tissues 24 h after treatment. In agreement with both our histological results and tissue swelling data, we also detected significantly higher IL‐6 and IFN‐*γ* expression in ConA‐stimulated tissue (Fig. 5). As IL‐6 stimulates and attracts neutrophils to the site of inflammation (Fielding et al. 2008; Scheller et al. 2011) and IFN‐*γ* controls trafficking of both neutrophils and macrophages (McLoughlin et al. 2003), our results indicate not only stronger leukocyte infiltration but also increased cellular activation and signaling following ConA injection.

In this study, we confirmed ConA as a potent and convenient pro‐inflammatory stimulant for use in the skin‐swelling test. In mammals, ConA induces stronger cellular activation of cytokine expression and a stronger tissue swelling response than PHA. During ConA treatment, we observed a significant positive relationship between magnitude of tissue swelling and tissue cellularity (mainly number of infiltrating neutrophils). This association between skin‐swelling magnitude and immune response intensity can be seen as a relationship between an externally measured trait important for ecological field research and a biologically meaningful underlying immune response mechanism, one of the basic assumptions of the skin‐swelling test. This association has been found repeatedly in studies measuring PHA‐induced skin swelling in birds (Martin et al. 2006; Vinkler et al. 2012) but remains unsupported in mammal studies (Turmelle et al. 2010; Merlo et al. 2014a). Our study also indicated no relationship between tissue cellularity and swelling magnitude following PHA stimulation in mice; hence, the PHA skin‐swelling test cannot be interpreted unambiguously and the mechanism responsible for induction and development of skin swelling in response to PHA in mammals remains unclear. In contrast, the clear positive relationship between skin‐swelling magnitude and cellular infiltration into tissue following ConA stimulation (both peaking 24 h after injection) resolves the basic immunological mechanism behind the skin‐swelling test outlined above. Hence, while PHA is currently the most‐widely used pro‐inflammatory stimulant for the skin‐swelling test (Kennedy and Nager 2006), we propose that ConA be used in all future ecological testing in mammals as it exhibits better performance and its application facilitates immunological interpretation of skin‐swelling test results. Based on the present knowledge of immunological mechanism underlying the response, we may confidently assume that our results and conclusions are generally valid for rodents and most other mammalian species: laboratory, domestic, and wild. We are aware of no biological reason why general features of the immune response should differ between the laboratory mouse we used in this study and free‐living animals that are investigated in ecological field research, conditioned that the architecture of their immune system is related. Note, however, that the stronger lymphocyte response induced by ConA appears to be taxon‐specific on large evolutionary scale. In sea bass (*Dicentrarchus labrax*) and loggerhead sea turtles (*Caretta caretta*), for example, PHA has been reported as inducing a stronger proliferation of blood‐derived lymphocytes than ConA (Rousselet et al. 2013; Ortiz et al. 2014). Therefore, our conclusions deserve further verification if applied on nonmammalian species.

## Conflict of Interest

None declared.

## Appendix

**Table A1:** Cellular infiltrate composition after ConA and PHA treatment. Cell counts are shown as mean number of cells in 100 *μ*m^2^ of tissue sample ± standard error. Results of Tukey tests (difference in infiltration between time points) are marked with superscript letters: ^a^significantly different from the control (time point 0), ^b^significantly different from 12 h, ^c^significantly different from 24 h. In all cases *P* < 0.05

Time (h)	Treatment	Total cell count	Lymphocytes	Neutrophils	Macrophages
	DPBS	0.19 ± 0.04	0.03 ± 0.01	0.13 ± 0.04	0.03 ± 0.01
0	—	0.04 ± 0.006	0.01 ± 0.003	0.02 ± 0.006	0.02 ± 0.01
3	ConA	0.36 ± 0.06^a,b,c^	0.07 ± 0.03	0.23 ± 0.05^b,c^	0.06 ± 0.02
6	ConA	0.48 ± 0.07^a,b,c^	0.11 ± 0.01	0.31 ± 0.06^a,b,c^	0.06 ± 0.02
12	ConA	0.79 ± 0.04^a^	0.12 ± 0.04	0.58 ± 0.04^a^	0.07 ± 0.02
24	ConA	0.84 ± 0.07^a^	0.1 ± 0.03	0.66 ± 0.05^a^	0.07 ± 0.02
48	ConA	0.44 ± 0.06^a,b,c^	0.07 ± 0.03	0.28 ± 0.04^a,b,c^	0.06 ± 0.02
72	ConA	0.43 ± 0.08^a,b^	0.04 ± 0.01	0.28 ± 0.05^a,b,c^	0.1 ± 0.02^a^
3	PHA	0.4 ± 0.05	0.06 ± 0.02	0.29 ± 0.05	0.06 ± 0.01
6	PHA	0.31 ± 0.03	0.07 ± 0.03	0.19 ± 0.02	0.05 ± 0.02
12	PHA	0.46 ± 0.06^a^	0.07 ± 0.01	0.31 ± 0.03	0.06 ± 0.03
24	PHA	0.46 ± 0.12^a^	0.04 ± 0.01	0.35 ± 0.10^a^	0.06 ± 0.02
48	PHA	0.6 ± 0.12^a^	0.08 ± 0.02	0.41 ± 0.08^a^	0.07 ± 0.03
72	PHA	0.48 ± 0.06^a^	0.09 ± 0.02	0.33 ± 0.06^a^	0.06 ± 0.02

**Table A2:** Full models tested and their levels of significance. Swelling PHA/ConA = magnitude of swelling following PHA/ConA treatment (thickness change); number of lymphocytes/neutrophils/macrophages PHA/ConA = number of cells infiltrating tissue following PHA/ConA treatment; time = time after treatment; age = age of animal; weight = weight of animal

Full model	*F*	DF	*P*
Swelling PHA ~ time + age + weight	26.64	8/26	<0.001
Swelling ConA ~ time+ age + weight	17.63	8/26	<0.001
Total cellular infiltration PHA ~ time + age + weight	4.18	8/26	0.003
Number of lymphocytes PHA ~ time + age + weight	2.97	8/26	0.017
Number of neutrophils PHA ~ time + age + weight	2.98	8/26	0.017
Number of macrophages PHA ~ time + age + weight	2.70	8/26	0.220
Total cellular infiltration ConA ~ time + age + weight	13.4	8/26	<0.001
Number of lymphocytes ConA ~ time + age + weight	4.79	8/26	0.001
Number of neutrophils ConA ~ time + age + weight	14.08	8/26	<0.001
Number of macrophages ConA ~ time + age + weight	4.67	8/26	0.001
